# Exploring the gap between coverage, access, and utilization of long-lasting insecticide-treated nets (LLINs) among the households of malaria endemic districts in Bangladesh

**DOI:** 10.1186/s12936-018-2610-0

**Published:** 2018-12-06

**Authors:** Fouzia Khanam, Md Belal Hossain, Tridib Roy Chowdhury, Md Sajedur Rahman, Moktadir Kabir, Shamsun Naher, Md Akramul Islam, Mahfuzar Rahman

**Affiliations:** 1 Research and Evaluation Division, BRAC, BRAC Centre, 75 Mohakhali, Dhaka, 1212 Bangladesh; 2 Tuberculosis and Malaria Control Programme, BRAC, BRAC Centre, 75 Mohakhali, Dhaka, 1212 Bangladesh

**Keywords:** Long-lasting insecticide net (LLIN), LLIN use, LLIN coverage, LLIN access, Malaria control programme, Bangladesh

## Abstract

**Background:**

Malaria is still a major public health concern in Bangladesh in spite of mass distribution of long-lasting insecticide-treated nets (LLINs) as a key preventive strategy. There might be a considerable gap between coverage and actual use of nets by the population in endemic areas. This study intended to assess the gap between coverage, access to and use of LLINs among the households in malaria-endemic settings in Bangladesh.

**Methods:**

This cross-sectional study collected data from 2640 households of 13 endemic districts of Bangladesh through three-stage cluster random sampling. The gap between coverage, access and use of LLINs were calculated using the procedure established by the Roll Back Malaria Monitoring and Evaluation Reference Group. To support the quantitative findings, qualitative data were also collected through in-depth interview, focus group discussion and key informant interview and analysed accordingly.

**Results:**

Of 2640 total households, 77.4% (n = 2044) possessed at least two LLINs, 56.8% (n = 1499) had insufficient access, and 18.8% (n = 495) had excess LLINs. Members of 77.9% (n = 2056) households had used LLINs the previous night and 6.0% (n = 68) did not use LLINs despite having sufficient access. LLIN use was lower in non-hill track areas, in Bengali community, in richer households and households with more than four members. Moreover, qualitative findings revealed that the major reasons behind not using LLINs were insufficient access, sleeping outside the home, migration, perceived low efficacy of LLINs, or fear of physical side effects.

**Conclusion:**

Closing the access gap by providing enough nets through solid investment and well-designed behavioural change interventions are crucial for achieving and sustaining universal coverage.

## Background

Although there has been significant success in the fight against malaria over the last few decades, malaria remains a major public health and socio-economic burden [1]. Globally, malaria accounted for 445,000 deaths in 2016 and is highly concentrated in the world’s poorest countries. In 2016, an estimated 216 million cases of malaria occurred worldwide. Most malaria cases in 2016 were in the World Health Organization (WHO) African region (90%), followed by the WHO Southeast Asian region (7%) and the WHO Eastern Mediterranean region (2%) [1]. Bangladesh, a country in South Asia, was also endemic for malaria, while the disease is now restricted to 71 *upazilas* of 13 districts of the country. Nearly 80% of total deaths attributed to malaria occurred in these 13 districts in Bangladesh [2]. Also, 90% of all malaria cases in Bangladesh are found in these 13 districts [2]. Currently, approximately 17.5 million people are at risk of becoming infected with malaria in those districts [3].

The distribution of long-lasting insecticide-treated bed nets (LLINs) is one of the key intervention strategies for preventing malaria in Bangladesh [4]. Increasing the coverage and use of LLINs is also the most preferred malaria vector control strategy in malaria-endemic countries, according to WHO recommendations [5]. Therefore, the national malaria control programme of Bangladesh has planned to scale up the distribution of LLINs to 100% and increase coverage (to at least two LLINs per household) in malaria-endemic areas, and to eliminate malaria by 2030 [3]. The Government of Bangladesh and an NGO consortium led by Building Resources Across Communities (BRAC) has been implementing the Global Fund to Fight AIDS, Tuberculosis and Malaria (GFATM)-funded malaria control programme since 2007 [6]. Up to now, Bangladesh has maintained a highly cost-effective insecticide-treated net coverage compared to other malaria-endemic countries [7–9]. The Government has to abide by the Global Technical Strategy for Malaria (2016–2030) to attain the long-term goal of worldwide malaria elimination and eventual eradication.

According to WHO recommendation, one LLIN should be distributed for every two people at risk of malaria to ensure universal access [1]. Since there is a high correlation between access and use of LLINs, to eliminate malaria, improving access to LLINs should be the major priority [10]. Globally, two main indicators were being used to assess LLINs use: the “proportion of households owning at least one LLIN” and the “proportion of children under five and pregnant women sleeping under LLINs the previous night” [11]. Consistently, these indicators show a considerable gap between coverage and actual use of nets by vulnerable groups, i.e., children and pregnant women. But the gap between coverage and required access and utilization was not completely measurable by these indicators, and was a bottleneck for assessing the LLINs access and proper usage. Although behaviour-driven failure plays a key role, evidence has shown that the main reason for non-use is lack of access to a net [12].

To measure the access to LLINs in a more appropriate way, the Roll Back Malaria (RBM) campaign recommended two additional core LLIN indicators [13]. One is the “proportion of households with at least one LLIN for every two people (household access)” which allows an estimate of the coverage gap (households with no or insufficient LLINs) in a better way. However, affirmative knowledge suggests the gaps in coverage, access and use of LLINs have not yet been studied in Bangladesh. The present study hypothesized that there was an existing gap between the coverage, access and utilization of LLINs. Therefore, this study aimed to explore the gaps in terms of coverage, access and use of LLINs at the household level in 13 malaria-endemic districts in Bangladesh. The study further aimed to measure the current gap and to explore the reason behind the gap.

## Methods

### Study design

This study employed a cross-sectional design triangulated with qualitative research method. In this case, three-stage cluster random sampling of households was applied. In the first stage of sampling, 30 *upazilas* were randomly selected from 71 *upazilas* where LLINs have been distributed. In the second stage of sampling, four villages were randomly selected from each *upazila* for statistically reliable estimate. In the third stage, 22 households per village having at least one under 5 and/or pregnant woman in the household were randomly selected.

### Study setting

**Fig. 1:**
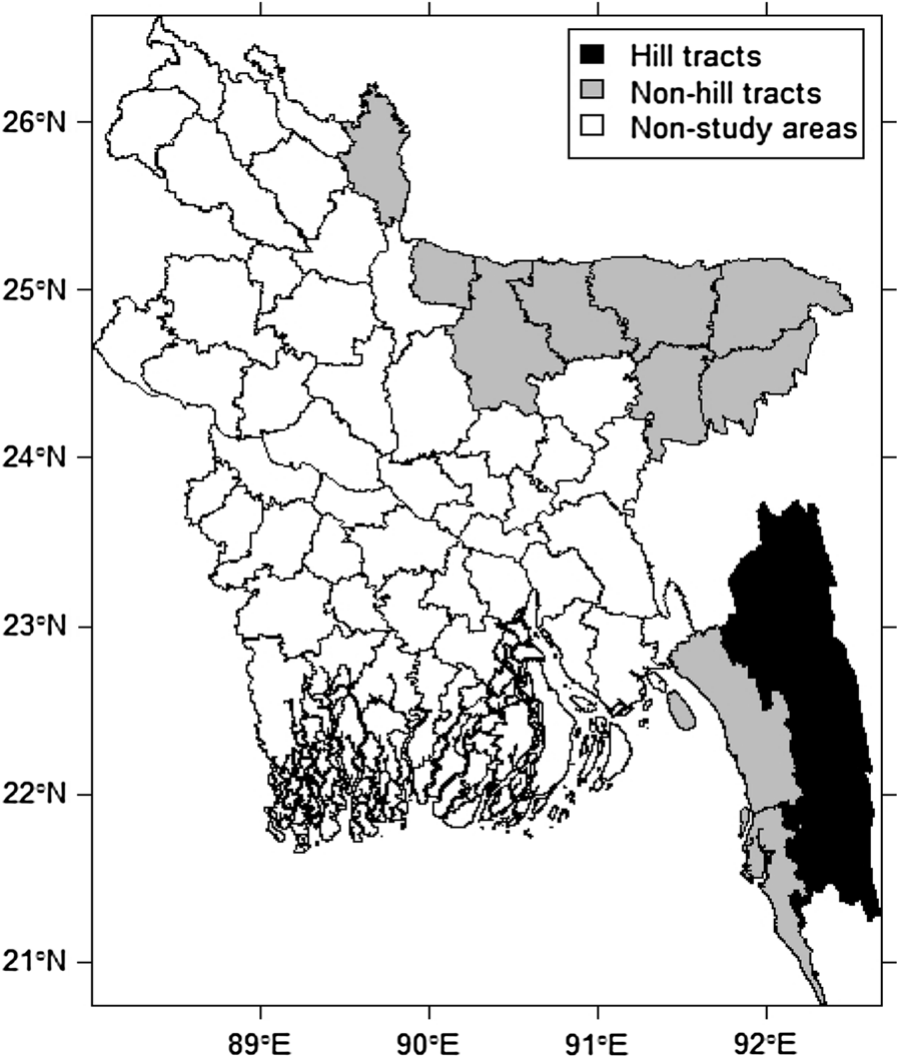
Map of the study area

This study was conducted in 13 malaria-endemic districts in the north, northeast and southeast regions in Bangladesh bordering India and Myanmar (Fig. 1), which included Khagrachhari, Rangamati, Bandarban, Cox’s Bazar, Chittagong, Sylhet, Sunamganj, Moulvibazar, Hobiganj, Mymensingh, Netrakona, Sherpur, and Kurigram. Three hill districts: Khagrachhari, Rangamati and Bandarban, located in the hilly remote areas of Chittagong hill track (CHT), suffer from a geographical disadvantage with difficult communication and intense perennial transmission of malaria. The field data were collected between April and May 2017.

### Participants

**Fig. 2:**
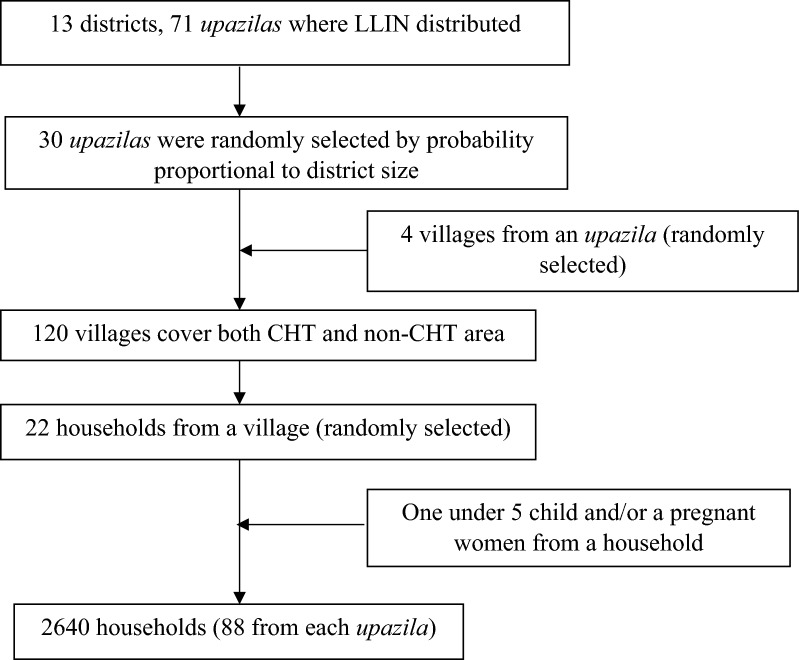
Flow chart of study area and sampling

Information on socio-economic and demographic condition, coverage, access to and use of LLINs was collected from 2640 households. The respondents were mainly the household head. If the household head was not present at the time of the interview, the information was collected from the husband/wife of household head or a pregnant woman or mother of children under 5 years old (Fig. 2).

For the qualitative part, a purposive sampling strategy was employed to select information-rich cases associated with the field of interest [14]. Two districts from two different endemic areas (CHT and non-CHT) and two villages from each district were selected. In each village, four in-depth interviews (with pregnant women) and one focus group discussion (with community people) were carried out. Another focus group discussion was conducted in each village with high-risk population groups, e.g., *Jhum* (cereal crop that grows in hills) cultivators, wood cutter and coal mine workers, and migrants as they often have limited access to prevention, diagnostic testing and treatment services. Three key-informant interviews were undertaken with frontline development workers of BRAC for qualitative exploration from each district.

### Data collection tools and techniques

Data were collected by research assistants using a pre-tested, structured questionnaire during face-to-face interviews with respondents. Research assistants were trained prior to data collection, encompassing lectures on completing the questionnaires, mock interviews, and field practice. A multilayered monitoring system was in place to validate, standardize and maintain quality of data and tasks such as spot checking, back checking, and provided necessary feedback to the teams working in the field. Different types of checklists were used in different data collection tools for qualitative segment. A team of four temporarily recruited research assistants with anthropology background, along with the lead qualitative researcher to collect field data.

### Variables assessed

The background variables for respondents were: age (< 20, 20–29 and ≥ 30 years), gender, relationship to household head (head, spouse, other), and level of education (no education, completed grade 1–5, completed grade 6–9, completed grade 10 or more). The background variables for household heads were: gender, level of education (no education, completed grade 1–5, completed grade 6–9, completed grade 10 or more), occupation (farmer, service holder, businessman, self-employed, labourer, housewife, others). The household level characteristics were: area (CHT, non-CHT), ethnicity (Bengali, others), religion (Muslim, Hindu, Buddhist, Christian, others), household size (≤ 4, > 4), wealth quintile (low, middle, high), having at least one child under 5 (yes/no), having at least one pregnant woman (yes/no).

Construction of the wealth index was based on factor analysis [15, 16] of key socio-economic variables. The key socio-economic variables were: types of wall, floor and roof of the house, ownership of radio, television, computer, bicycle, mobile/telephone, refrigerator, wardrobe, table, chair, watch, bed, sewing machine, bike, motor vehicle, livestock, and access to solar system, electricity.

### Outcome variables

Household-based indicators were used for assessment of coverage and access of LLINs. LLIN use was recorded as whether the household member/s slept under it the night prior to the day of survey [17].

#### Proportion of households with at least one (or two) LLINs (P_1_)

The numerator consists of all households having at least two LLINs and the denominator is the total number of sampled households.

#### Proportion of households with at least one (or two) usable LLINs (P_2_)

The numerator consists of all households having at least two usable (no visible rift on the net) LLINs and the denominator is the total number of sampled households.

#### Proportion of households with access to LLINs (P_3_)

The numerator contains all households where the ratio between numbers of LLINs owned and the numbers of household members is 0.5 or higher while the denominator is the total number of sampled households. This indicator is also defined as households with at least one LLIN for every two people.

#### Proportion of households with access to LLINs if any LLINs (P_4_)

The numerator contains all households where the ratio between numbers of LLINs owned and the numbers of household members is 0.5 or higher while the denominator is the total number of sampled households having at least one LLIN.

#### Proportion of households uses LLINs the previous night (P_5_)

The numerator contains all households whose members slept under LLINs the previous night and the denominator is the total households in the sample.

#### Proportion of households that used LLINs the previous night if accessed (P_6_)

The numerator contains all households whose members slept under LLINs the previous night and the denominator is the total households having sufficient access to LLINs.

#### Access gap

Households not having at least one LLIN for every two people, i.e., 1 − P_3_, are defined as having insufficient access to LLINs or access gap.

#### Intra-household net gap

Household that did not have access to LLINs despite possessing LLINs, i.e., 1 − P_4_, is defined as intra-household net gap.

#### Use gap

Household that did not use LLINs the previous night despite having access, 1 − P_6_, is defined as the use gap of LLINs.

### Statistical analysis

Descriptive analyses were conducted to show the distribution of different characteristics of the respondents and household heads with appropriate cut-offs. The Chi square test [18] was done to assess association of different background characteristics with LLIN coverage, access and use. Binary logistic regression [19] was applied to assess relationship between background characteristics and LLIN use. The final model was adjusted with area of residence, gender and education of household head, ethnicity, religion, household size, wealth quintile, status of household having at least one child under 5 years, and having at least one pregnant woman, and with respondents’ relationship to household head. The adjusted odds ratio (AOR), exponent of beta-coefficient of binary logistic regression was calculated with 95% confidence interval (95% CI). All tests were done at 5% level of significance. All analyses were performed using STATA software (Version 13.0, STATA Corp LP, TX, USA).

## Results

### Background characteristics

Information was collected from a total of 2640 households, where one-third was from CHT area and remaining from non-CHT area. The average family size was 5.5 ± 2.2, with 1.06 female to male ratio. The majority of the respondents were females (88.8%), with 20 or more years of age (91.7%). Only 13.8% respondents were household heads. Nearly one-third of respondents had no formal education and only 11.7% had completed grade 10 or more. In terms of ethnicity and religion, 79.1% were Bengali and 70.3% were Muslim. Also, 60.1% households had more than four members, 76% households had at least one child under 5 and 46.1% households had at least one pregnant woman (Table 1).

**Table 1:** Background characteristics of respondents and household heads (N = 2640)

Characteristics	n	%	95% CI
Area of residence			
Hill tract	880	33.33	31.56–35.16
Non-hill tract	1760	66.67	64.84–68.44
Gender of respondent			
Male	296	11.21	10.06–12.47
Female	2344	88.79	87.53–89.94
Age of respondent (years)			
< 20	218	8.26	7.27–9.37
20–29	1379	52.23	50.33–54.14
30+	1043	39.51	37.66–41.39
Relationship to household head			
Head	363	13.75	12.49–15.12
Spouse	1885	71.40	69.65–73.09
Others^a^	392	14.85	13.54–16.26
Education of respondent			
No education	583	22.08	20.54–23.71
1–5	917	34.73	32.94–36.57
6–9	819	31.02	29.29–32.82
10+	321	12.16	10.97–13.46
Gender of household head			
Male	2410	91.29	90.15–92.31
Female	230	8.71	7.69–9.85
Education of household head			
No education	858	32.50	30.74–34.31
1–5	942	35.68	33.87–37.53
6–9	531	20.11	18.63–21.69
10+	309	11.70	10.53–12.99
Occupation of household head			
Farmer	677	25.64	24.01–27.35
Service holder	261	9.89	8.80–11.09
Businessman	481	18.22	16.79–19.74
Self-employment	85	3.22	2.61–3.97
Labourer	892	33.79	32.01–35.62
Housewife	143	5.42	4.61–6.35
Others^b^	101	3.83	3.16–4.63
Ethnicity			
Bengali	2089	79.13	77.54–80.64
Others	551	20.87	19.36–22.46
Religion			
Muslim	1857	70.34	68.57–72.05
Hindu	222	8.41	7.41–9.53
Buddhist	488	18.48	17.05–20.01
Christian	51	1.93	1.47–2.53
Others^c^	22	0.83	0.55–1.26
Number of household members			
≤ 4	1054	39.92	38.07–41.81
> 4	1586	60.08	58.19–61.93
Wealth quintile			
Low	1059	40.11	38.26-42.00
Middle	525	19.89	18.41–21.45
High	1056	40.00	38.15–41.88
Household having at least one child under five			
No	634	24.02	22.42–25.68
Yes	2006	75.98	74.32–77.58
Household having at least one pregnant woman			
No	1422	53.86	51.96–55.76
Yes	1218	46.14	44.24-–48.04
N	2640	100.00	

^a^Son, daughter, father, mother, brother, father-in-law, mother-in-law

^b^Student, disabled, beggar

^c^Garo, Chakma, Marma, Tripura, Mro, Hajong, Sawtal, Tongchonga, Bomo

### Coverage of LLINs

A total of 6048 LLINs were distributed among 2640 households with a mean 2.3 ± 1.1 LLINs per households. At the time of the survey, almost every household had at least one LLIN (99.8%), 93.1% households had at least one useable LLIN, 77.4% households had at least two LLINs, and 58.4% households had at least two useable LLINs (Table 2). The coverage of LLINs was high in hill track areas (*P *= 0.003 for at least two LLINs), households with female heads (*P *= 0.040 for at least two usable LLINs), and in rich households (*P *< 0.001 for at least two LLINs or at least two usable LLINs).

**Table 2:** LLIN coverage by selected background characteristics

Characteristics	N	HHs at least with 1 LLIN		HHs at least with 1 usable LLIN		HHs at least with 2 LLINs (P_1_)		HHs at least with 2 usable LLINs (P_2_)	
		% (95% CI)	*P*	% (95% CI)	*P*	% (95% CI)	*P*	% (95% CI)	*P*
Area of residence									
Hill tract	880	99.89 (99.20–99.98)	0.527	94.66 (92.96–95.96)	0.023	80.91 (78.18–83.37)	0.003	58.75 (55.46–61.96)	0.802
Non-hill tract	1760	99.77 (99.40–99.91)		92.27 (90.93–93.43)		75.68 (73.62–77.63)		58.24 (55.92–60.52)	
Gender of household head									
Male	2410	99.79 (99.50–99.91)	0.489	93.11 (92.03–94.06)	0.774	77.01 (75.29–78.65)	0.102	57.80 (55.82–59.76)	0.040
Female	230	100.00		92.61 (88.43–95.36)		81.74 (76.21–86.22)		64.78 (58.39–70.69)	
Education of household head									
No education	858	99.65 (98.92–99.89)	0.426	91.96 (89.94–93.60)	0.401	79.14 (76.29–81.73)	0.140	58.86 (55.53–62.11)	0.095
1–5	942	99.79 (99.15–99.95)		93.52 (91.76–94.93)		75.27 (72.41–77.92)		55.84 (52.65–58.98)	
6–9	531	100.00		93.22 (90.74–95.07)		76.84 (73.05–80.23)		59.13 (54.89–63.24)	
10+	309	100.00		94.50 (91.33–96.55)		80.26 (75.44–84.33)		63.75 (58.24–68.93)	
Number of household members									
≤ 4	1054	99.62 (98.99–99.86)	0.067	89.56 (87.57–91.27)	< 0.001	55.22 (52.20–58.20)	< 0.001	37.95 (35.07–40.92)	< 0.001
> 4	1586	99.94 (99.55–99.99)		95.40 (94.25–96.33)		92.18 (90.75–93.41)		72.01 (69.74–74.16)	
Wealth quintile									
Low	1059	99.62 (99.00–99.86)	0.136	92.16 (90.38–93.64)	0.196	71.95 (69.17–74.58)	< 0.001	49.86 (46.85–52.87)	< 0.001
Middle	525	99.81 (98.66–99.97)		92.76 (90.21–94.69)		78.29 (74.55–81.61)		58.86 (54.59–63.00)	
High	1056	100.00		94.13 (92.54–95.40)		82.48 (80.07–84.66)		66.76 (63.86–69.54)	
Overall	2640	99.81 (99.55–99.92)		93.07 (92.03–93.98)		77.42 (75.79–78.98)		58.41 (56.52–60.28)	

### Access to LLINs

Only 43.2% households had sufficient access to LLINs at the time of survey (Table 3). Household access to LLINs was significantly higher in hill track area (54.1 vs 37.8%, *P *< 0.001), in households with female head (50.4 vs 42.5%, *P *= 0.021) and in households with 4 or fewer members (63 vs 30.8%, *P *< 0.001). Furthermore, LLIN access increased with an increase in household head’s level of education.

**Table 3:** Access to LLINs by selected background characteristics

Characteristics	N	HHs with access to LLINs (P_3_)		N	HHs with access to LLINs if any LLINs (P_4_)	
		% (95% CI)	*P*		% (95% CI)	*P*
Area of residence						
Hill tracts	880	54.09 (50.78–57.36)	< 0.001	879	54.15 (50.84–57.43)	< 0.001
Non-hill tracts	1760	37.78 (35.55–40.08)		1756	37.87 (35.63–40.17)	
Gender of household head						
Male	2410	42.53 (40.57–44.52)	0.021	2405	42.62 (40.65–44.61)	0.022
Female	230	50.43 (44.56–56.86)		230	50.43 (44.00–56.86)	
Education of household head						
No education	858	39.28 (36.06–42.59)	0.001	855	39.42 (36.19–42.74)	0.002
1–5	942	42.46 (39.34–45.65)		940	42.55 (39.42–45.74)	
6–9	531	46.33 (42.12–50.59)		531	46.33 (42.12–50.59)	
10+	309	51.13 (45.56–56.67)		309	51.13 (45.56–56.67)	
Number of household members						
≤ 4	1054	63.00 (60.04–65.86)	< 0.001	1050	63.24 (60.27–66.10)	< 0.001
> 4	1586	30.08 (27.87–32.38)		1585	30.09 (27.88–32.40)	
Wealth quintile						
Low	1059	43.44 (40.48–46.44)	0.890	1055	43.60 (40.63–46.62)	0.888
Middle	525	42.29 (38.12–46.56)		524	42.37 (38.20–46.65)	
High	1056	43.47 (40.50–46.48)		1056	43.47 (40.50–46.48)	
Overall	2640	43.22 (41.34–45.12)		2635	43.30 (41.42–45.20)	

### Use of LLINs

**Fig. 3:**
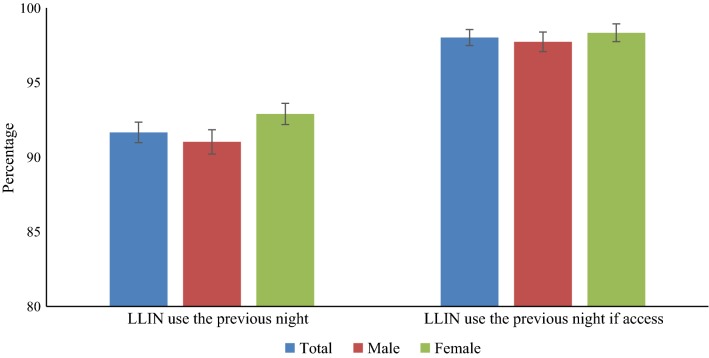
Proportion of people who used LLIN in the previous night

Out of a total 14,475 members in 2640 households, 91.7% slept under an LLIN the previous night. LLIN use was higher among females than males (92.9 vs 91.0%), and the use of LLINs among females than males increased if the household met with sufficient access (98.3 vs 97.7%) (Fig. 3).

The proportion of LLIN use by households was 77.9%, which means all the members of those households slept under an LLIN the previous night (Table 4). The households’ use of LLINs was higher in hill track areas than non-hill track areas (86.4 vs 73.4%, *P *< 0.001), and in households with 4 or fewer members than those with more than 4 members (90 vs 69.8%, *P *< 0.001). Although LLIN coverage was high, usage was reportedly low in rich households (74.2%). Moreover, households’ use of LLINs increased when they had sufficient access to LLINs (94%) (Table 4). This phenomenon is also reported in the qualitative part of the study. According to the qualitative analysis, the lack of access to LLINs was one of the most documented reasons for low utilization. Sleeping outdoors, migrating outside, feeling uncomfortable, lack of knowledge, incompatibility with sleeping arrangements, saving LLINs for another time, and perceived degraded efficacy of the nets were also reported as reasons for poor utilization of LLINs (Table 5).

**Table 4:** Use of LLINs by selected background characteristics

Characteristics	N	HHs use LLINs the previous night (P_5_)		N	HHs use LLINs the previous night if any LLINs		N	HHs use LLINs the previous night if access (P_6_)	
		% (95% CI)	*P*		% (95% CI)	*P*		% (95% CI)	*P*
Area of residence									
Hill tracts	880	86.36 (83.93–88.48)	< 0.001	879	86.46 (84.04–88.57)	< 0.001	476	95.38 (93.08–96.94)	0.107
Non-hill tracts	1760	73.64 (71.53–75.64)		1756	73.80 (71.69–75.81)		665	93.08 (90.88–94.78)	
Gender of household head									
Male	2410	78.22 (76.52–79.82)	0.177	2405	78.38 (76.69–79.98)	0.159	1025	94.63 (93.07–95.86)	0.012
Female	230	74.35 (68.31–79.58)		230	74.35 (68.31–79.58)		116	88.79 (81.64–93.39)	
Education of household head									
No education	858	76.11 (73.14–78.84)	0.076	858	76.37 (73.41–79.10)	0.097	337	95.25 (92.39–97.07)	0.165
1–5	942	77.18 (74.38–79.75)		940	77.34 (74.55–79.91)		400	92.75 (89.76–94.92)	
6–9	531	81.92 (78.41–84.97)		531	81.92 (78.41–84.97)		246	95.93 (92.60–97.80)	
10+	309	77.99 (73.03–82.27)		309	77.99 (73.03–82.27)		158	91.77 (86.34–95.17)	
Number of household members									
≤ 4	1054	90.04 (88.08–91.71)	< 0.001	1050	90.38 (88.44–92.02)	< 0.001	664	96.54 (94.84–97.69)	< 0.001
> 4	1586	69.80 (67.49–72.01)		1585	69.84 (67.53–72.05)		477	90.57 (87.59–92.89)	
Wealth quintile									
Low	1059	81.87 (79.43–84.08)	< 0.001	1055	82.18 (79.75–84.37)	< 0.001	460	94.78 (92.33–96.48)	0.125
Middle	525	77.14 (73.35–80.54)		524	77.29 (73.50–80.68)		222	95.95 (92.39–97.88)	
High	1056	74.24 (71.52–76.79)		1056	74.24 (71.52–76.79)		459	92.37 (89.56–94.48)	
Overall	2640	77.88 (76.25–79.42)		2635	78.03 (76.40–79.57)		1141	94.04 (92.51–95.28)

**Table 5:** Barriers against LLIN use as identified through focus group discussions, in-depth interviews, and key informants interviews

Insufficient access to LLINs within households: Some participants complained they do not have sufficient number of LLINs for all household members *“I have four beds in my household but got only one LLIN, how will we prevent ourselves from having malaria”* (FGD, Netrokona)
Shape of the net: Given the rectangular nature of the nets, it is not comfortable to hang over sleeping area, ‘incompatibility with sleeping arrangements and house style’
Sleeping outdoor: Many FGD and key informants mentioned that the family members who slept outdoors for work (especially woodcutter, zoom cultivator) cannot always use LLINs
Migration: The study documented some people who migrated in Bandarban district and those who are facing a shortage of LLINs. So that they mostly use normal mosquito nets or coils to avoid mosquito bites at night. SK of Bandarban told us that there are some families and also single individuals who regularly migrate from rural to urban areas. Some of the families came before survey and some not, but only the families who come before survey can get LLINs. Individuals living in mess/shared rooms for the purpose of education or service were not usually counted as a programme beneficiary
Perceived low efficacy of LLINs: Some participants argued that mosquito net is not of good quality as before. One of the participants mentioned *“The LLINs we received previously from BRAC was of very good quality indeed; once we hanged those nets there we couldn’t find any mosquito in that whole room but now using this LLINs we found poor quality from our experiences. This net is more or less similar with an ordinary mosquito net because we found mosquito with it even after hanging before sleeping.”* [FGD participant]
Fear of physical problems: Some may have faced various physical problems while using LLINs: burning inside eye, body itching and dyspnea were major physical problems quoted by participants in FGD sessions. According to the statements of those who had faced such problems never used LLIN in their household for a long time. One also reported a household who received LLINs in 2014. During the first time they were not very aware of usage procedure of LLINs and found a burning sensation inside eye as a side effect of LLINs. So they moved the mosquito net and started using coil instead. Another example might show the problem of body itching and dyspnea during LLIN use. They also moved the mosquito net but they never tried nor understood to look for a solution *“Some people complained that they suffered from burning eyes, swelling of the eye after using LLINs. Some people complained that it has bad smell”* (SS, Kolmakanda)
Saving LLIN for another time: People mentioned that they saved the nets for someone *“A mother kept her LLIN for her son. Her son works in India and often comes home. So she and her daughter are using the normal bed nets, saving the LLIN for her son*”
Lack of knowledge. Some people do not use LLINs because of their lack of knowledge regarding the importance of LLINs *“Some people do understand. I have seen some family that pregnant mothers or under five children are sleeping under normal bed nets, whereas other family members are using LLINs. Because they do not understand the difference between LLINs and normal bed nets”* (SS, Kolmakanda)
Uncomfortable: another reason for not using LLINs “*I am pregnant and I feel a sense of breathlessness whenever I use LLIN. Every time I hang LLIN I feel sick. So I use mosquito coil for safety.*” (IDI, pregnant women)
Presence of a guest: LLINs are distributed according to family members, so, if the family has a guest, some members have to sleep without LLINs “*We have four members in our family but we have only one mosquito net. In this situation my father*-*in*-*law might come someday and we only have one mosquito net. How would it be possible to manage the situation with one net? Though we received 1 mosquito net almost 3* *years ago and after that we received another one. But day after day those nets became quite obsolete to use so we put them underground.”* (IDI, Netrokona)

### LLIN coverage, access and use gaps

This study identified three broad areas of gaps in utilizing LLINs: intra-household net allocation, household inaccessibility, and proper usage of LLINs. Only 0.19% (5 out of 2640) households did not have LLINs at the time of the survey. Consequently, intra-household net gap was 56.7%, and 56.8% households had insufficient access. Contrarily, 18.8% (495 out of 2640) households had excess LLINs (i.e., more than one LLIN for every two people). Moreover, the use gap was 6%, meaning members from those 6% households who had sufficient access to LLINs did not use them the previous night.

The qualitative findings of the study also discovered some gap between coverage, access and utilization. Despite having large coverage in the respective locality, some people experienced a lack of LLINs as per the needs of family members. Daily life experience of Rasheda (pseudonym) of Netrokona district can illustrate this statement:

*“Having one LLIN in a family of four members is very crucial when necessity is not able to supplement. Moreover, we might face a situation in coming days as my father*-*in*-*law would possibly come. How would it be possible to manage the situation with only one net?”*

Another issue came to light regarding coverage gap which signifies that the mosquito nets were provided only among families. Individuals living separately in a mess or in shared rooms might not have received LLINs from the programme. SK from Bandarban adds:

*“Someone came to me one day while we were distributing mosquito net among the listed population. He asked for a net but we were not able to serve him as we do not have permission to give it to any individual who live in a mess or shared household or who was not in our distribution list. I felt pity for him but there was nothing I could do.”*

### Association between background characteristics and LLIN use

The association of background factors of households with sufficient and insufficient access to LLINs is shown in Table 6. Of households with sufficient access to LLINs, LLIN use was approximately five times less for Bengali community than others (AOR 0.22, 95% CI 0.07–0.66). Besides, households with more than four members had more than double likelihood of using LLINs than households with four or fewer members (AOR 2.64, 95% CI 1.51–4.59). For households with insufficient access to LLINs, the odds of using LLINs was approximately 50% higher in hill-track areas than non-hill track areas (AOR 1.46, 95% CI 1.04–2.04). Similarly, the Bengali community had lower odds of using LLINs (AOR 0.53, 95% CI 0.30–0.94), and households with more than four members had higher odds (AOR 2.13, 95% CI 1.60–2.82). Remarkably, households with high wealth quintiles were 24% less likely to use LLINs than poor households (AOR 0.76, 95% CI 0.57–0.99). Besides, the odds of LLIN use were nearly 50% higher when there was at least one child under five in the household (AOR 1.47, 95% CI 1.03–2.08). In addition, the odds of LLIN use was 60% higher for spouses compared to household heads (AOR 1.60, 95% CI 1.12–2.27) (Table 6).

**Table 6:** Association between background characteristics and LLINs use of previous night

Characteristics	HHs with sufficient access to LLINs			*P*	HHs with insufficient access to LLINs			
	LLINs use the previous night				LLINs use the previous night			
	No, n (%)	Yes, n (%)	AOR (95% CI)		No, n (%)	Yes, n (%)	AOR (95% CI)	*P*
Area of residence								
Non-hill tract	46 (6.92)	619 (93.08)	1.00		418 (38.17)	677 (61.83)	1.00	
Hill tract	22 (4.62)	454 (95.38)	0.88 (0.46–1.70)	0.707	98 (24.26)	306 (75.74)	1.46 (1.04–2.04)	0.028
Gender of household head								
Male	55 (5.37)	970 (94.63)	1.00		470 (33.94)	915 (66.06)	1.00	
Female	13 (11.21)	103 (88.79)	0.65 (0.3–1.42)	0.281	46 (40.35)	68 (59.65)	1.13 (0.73–1.74)	0.581
Education of household head								
No education	16 (4.75)	321 (95.25)	1.00		189 (36.28)	332 (63.72)	1.00	
1–5	29 (7.25)	371 (92.75)	0.57 (0.29–1.11)	0.097	186 (34.32)	356 (65.68)	1.02 (0.78–1.33)	0.890
6–9	10 (4.07)	236 (95.93)	0.92 (0.39–2.18)	0.870	86 (30.18)	199 (69.82)	1.30 (0.93–1.81)	0.128
10+	13 (8.23)	145 (91.77)	0.43 (0.18–1.00)	0.050	55 (36.42)	96 (63.58)	0.94 (0.62–1.41)	0.751
Ethnicity								
Others	10 (3.0)	323 (97.0)	1.00		46 (21.10)	172 (78.90)	1.00	
Bengali	58 (7.18)	750 (92.82)	0.22 (0.07–0.66)	0.007	470 (36.69)	811 (63.31)	0.53 (0.30–0.94)	0.029
Religion								
Others^a^	19 (4.58)	396 (95.42)	1.00		109 (29.62)	259 (70.38)	1.00	
Muslim	49 (6.75)	677 (93.25)	1.56 (0.71–3.41)	0.270	407 (35.99)	724 (64.01)	1.10 (0.77–1.57)	0.608
Number of household members								
≤ 4	23 (3.46)	641 (96.54)	1.00		82 (21.03)	308 (78.97)	1.00	
> 4	45 (9.43)	432 (90.57)	2.64 (1.51–4.59)	0.001	434 (39.13)	675 (60.87)	2.13 (1.60–2.82)	< 0.001
Wealth quintile								
Low	24 (5.22)	436 (94.78)	1.00		168 (28.05)	431 (71.95)	1.00	
Middle	9 (4.05)	213 (95.95)	2.11 (0.93–4.78)	0.072	111 (36.63)	192 (63.37)	0.79 (0.58–1.08)	0.143
High	35 (7.63)	424 (92.37)	1.21 (0.65–2.26)	0.546	237 (39.70)	360 (60.30)	0.76 (0.57–0.99)	0.045
Household having at least one child under five								
No	19 (4.75)	381 (95.25)	1.00		98 (41.88)	136 (58.12)	1.00	
Yes	49 (6.61)	692 (93.39)	0.84 (0.41–1.74)	0.644	418 (33.04)	847 (66.96)	1.47 (1.03–2.08)	0.033
Household having at least one pregnant woman								
No	33 (6.17)	502 (93.83)	1.00		292 (32.92)	595 (67.08)	1.00	
Yes	35 (5.78)	571 (94.22)	0.94 (0.49–1.81)	0.858	224 (36.60)	388 (63.40)	1.05 (0.80–1.37)	0.743
Relationship to household head								
Head	16 (9.47)	153 (90.53)	1.00		74 (38.14)	120 (61.86)	1.00	
Spouse	35 (4.30)	779 (95.70)	2.04 (0.99–4.20)	0.053	323 (30.16)	748 (69.84)	1.60 (1.12–2.27)	0.009
Others^b^	17 (10.76)	141 (89.24)	0.98 (0.44–2.19)	0.967	119 (50.85)	115 (49.15)	0.78 (0.52–1.18)	0.237

^a^Garo, Chakma, Marma, Tripura, Mro, Hajong, Sawtal, Tongchonga, Bomo

^b^Son, daughter, father, mother, brother, father-in-law, mother-in-law

## Discussion

The present study assessed the gap between LLIN coverage, access and utilization in 13 malaria-endemic districts in Bangladesh, based on newly recommended Monitoring and Evaluation Reference Group (MERG) indicators [13, 20]. Reported LLIN coverage is far better (99.8%) compared to previous year’s coverage [4], demonstrating continuous efforts by the government and other NGOs in taking initiatives to prevent malaria. However, the qualitative findings revealed that in urban settings, the migratory people, especially bachelors who live alone had low coverage. This might be as a result of targeted LLIN distribution campaigns which mainly focus on rural and family settings. To achieve universal coverage, LLIN distribution strategies need to be promoted to address the needs of urban as well as rural settings.

To achieve universal coverage, the goal is to ensure one LLIN for every two people, as per WHO’s recommendation. The present study identified that only 43.2% households had enough LLINs for every member of that household. However, examining those households who own any LLINs revealed that the coverage of at least one LLIN for every two people was 43.3%, while it was 54.2% in the hilly areas. The intra-household net gap was 56.7%, while the gap was 45.8% in the hilly areas. Even with fairly high coverage of at least one LLIN, nearly half of the households in highly endemic areas did not have enough access to LLINs. This suggested a huge gap between coverage and access in malaria-endemic settings, which would hamper universal coverage and the national target [5]. In contrast, 18.8% of households had extra LLINs, suggesting the inequitable distribution of LLINs in the community. These findings are consistent with other studies in a similar context [17, 21, 22]. Some households might have purchased more nets or saved some nets for times of necessity, which could be the reason for the presence of extra LLINs at the time of the survey. This was also corroborated in the qualitative part of this study.

In malaria-endemic settings, high utilization of LLINs is the central goal for the malaria control programme as LLINs are one of the most cost-effective interventions. The present study found that 77.9% of households used LLINs (all the members of those households slept under LLINs the previous night). The high utilization, in contrast to low access, indicates that more than two people were sharing a net. This was not surprising considering that children may be sharing both sleeping space and LLINs with their parents, especially in conditions where LLINs were scarce or in homes where hanging multiple nets was not possible due to limited sleeping space [23, 24].

Moreover, when households had sufficient access to LLINs their use increased to 94%, which indicates the difference between non-use due to lack of access and non-use due to behavioural failure. The vast majority of those who had access to LLINs were using them. This implies that non-use is mostly associated with lack of access, which is confirmed by the qualitative part of the study. And these findings are in line with other national level studies [10] which established that access is the key driver of LLIN use. Nevertheless, use of LLINs was higher in the hilly areas, in women and in households having at least one child under five, which reflects the success of extensive behaviour change communication (BCC) efforts in the past decade that encouraged LLIN use by vulnerable groups, such as women and children. These results should encourage both donors and malaria control programme officials that the effort and investment are not wasted. The malaria control programme should continue its efforts towards closing the access gap through continuous distribution of LLINs through community as well as through social marketing and retail sales.

The qualitative component of the study offers insights into the reason for not using LLINs. Outdoor sleeping practices, living alone in a mess, and migration were documented reasons for poor utilization of LLINs. Conventional LLIN promotion approaches that focus mostly on family and indoor interventions with an emphasis on women and children should be re-oriented and refocused. Several other reasons for not using LLINs were feeling uncomfortable, fear of physical problem, perceived low efficacy of LLINs, and lack of knowledge. Moreover, 6% of household members were not using LLINs even though they had enough, reflecting their behavioural failure, which demands targeted behavioural interventions.

### Study limitations

This survey was conducted following the rainy season in the study area when mosquitoes were abundant. This should be taken into account when interpreting the findings as the season might have encouraged people to use the nets more. Outcomes in coverage and access as well as LLIN utilization could show different proportions if the study was conducted in other seasons of the year. It is challenging to investigate causation from cross-sectional design, and only association can be truly estimated. A strength of this study is direct observation that has been conducted by data collectors and research staff for all applicable questions in order to minimize response bias during the interviews.

## Conclusion

Achieving universal coverage of LLINs was short of the targets, with a relatively wide intra-household net gap. Although higher access rate usually increases net use, there is still a gap between access and utilization. Therefore, moving towards elimination and eventual eradication, the malaria control programme needs to take into consideration three important aspects: (1) ensuring sufficient provision of nets to every household; (2) targeting specific population groups to achieve and maintain universal LLIN coverage; and, (3) concentrating on well-designed behavioural change interventions to resolve behaviour-driven non-use. To achieve and sustain universal user coverage of LLINs in malaria-endemic areas, solid investment and well-designed BCC interventions are mandatory.

## Abbreviations

AOR: adjusted odds ratio
BCC: behaviour change communication
BRAC: Building Resources Across Communities
CHT: Chittagong hill tract
CI: confidence interval
GFATM: Global Fund to Fight AIDS, Tuberculosis and Malaria
LLIN: long-lasting insecticide-treated net
MERG: Monitoring and Evaluation Reference Group
WHO: World Health Organization
